# (*R*)-2-(2-Methoxy­phen­yl)-2,5-dihydro­thio­phene-3-carbaldehyde

**DOI:** 10.1107/S1600536810000991

**Published:** 2010-01-13

**Authors:** Jie Tang, Ai-Bao Xia, Jun-Rong Jiang, Yifeng Wang

**Affiliations:** a State Key Laboratory Breeding Base of Green Chemistry–Synthesis Technology, Zhejiang University of Technology, Hangzhou 310014, People’s Republic of China

## Abstract

In the title compound, C_12_H_12_O_2_S, the asymmetric unit contains two independent mol­ecules. The chiral C atoms of both mol­ecules were established to be in the *R* configuration. In both mol­ecules, the 2,5-dihydro­thio­phene rings adopt *S*-envelope conformations wherein the S atoms are displaced by 0.315 (5) and −0.249 (5) Å from the mean planes of the remaining ring atoms. In the crystal, the molecules are linked by weak C—H⋯O interactions.

## Related literature

For background to the organocatalytic domino reaction, see: Enders *et al.* (2007 ▶); Yu & Wang (2008 ▶). For a related structure, see: Zhu *et al.* (2009 ▶).
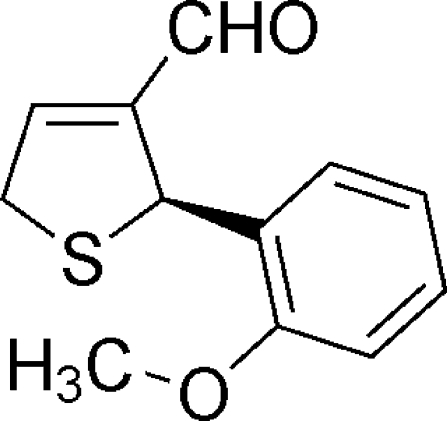


## Experimental

### 

#### Crystal data

C_12_H_12_O_2_S
*M* *_r_* = 220.28
Triclinic, 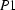

*a* = 6.8281 (6) Å
*b* = 7.7624 (9) Å
*c* = 11.8748 (12) Å
α = 71.733 (3)°
β = 79.051 (3)°
γ = 73.488 (3)°
*V* = 569.50 (10) Å^3^
*Z* = 2
Mo *K*α radiation
μ = 0.26 mm^−1^
*T* = 296 K
0.46 × 0.42 × 0.38 mm

#### Data collection

Rigaku R-AXIS RAPID diffractometer
Absorption correction: multi-scan (*ABSCOR*; Higashi, 1995 ▶) *T*_min_ = 0.886, *T*_max_ = 0.905
5623 measured reflections
4243 independent reflections
3098 reflections with *I* > 2σ(*I*)
*R*_int_ = 0.031

#### Refinement

*R*[*F* ^2^ > 2σ(*F* ^2^)] = 0.039
*wR*(*F* ^2^) = 0.116
*S* = 1.00
4243 reflections
274 parameters
3 restraints
H-atom parameters constrained
Δρ_max_ = 0.25 e Å^−3^
Δρ_min_ = −0.31 e Å^−3^
Absolute structure: Flack (1983 ▶), 1657 Friedel pairs
Flack parameter: 0.02 (9)

### 

Data collection: *PROCESS-AUTO* (Rigaku, 2006 ▶); cell refinement: *PROCESS-AUTO*; data reduction: *CrystalStructure* (Rigaku, 2007 ▶); program(s) used to solve structure: *SHELXS97* (Sheldrick, 2008 ▶); program(s) used to refine structure: *SHELXL97* (Sheldrick, 2008 ▶); molecular graphics: *ORTEP-3 for Windows* (Farrugia, 1997 ▶); software used to prepare material for publication: *WinGX* (Farrugia, 1999 ▶).

## Supplementary Material

Crystal structure: contains datablocks global, I. DOI: 10.1107/S1600536810000991/pv2254sup1.cif

Structure factors: contains datablocks I. DOI: 10.1107/S1600536810000991/pv2254Isup2.hkl

Additional supplementary materials:  crystallographic information; 3D view; checkCIF report

## Figures and Tables

**Table 1:** Hydrogen-bond geometry (Å, °)

*D*—H⋯*A*	*D*—H	H⋯*A*	*D*⋯*A*	*D*—H⋯*A*
C7*A*—H7*A*⋯O1*B*	0.93	2.60	3.473 (4)	157
C4*B*—H4*B*1⋯O1*B*^i^	0.97	2.69	3.202 (4)	113

Symmetry code: (i) 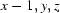.

**Fig. 1.**
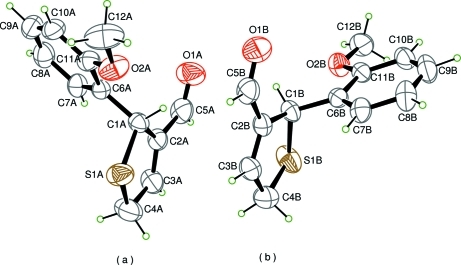
An ORTEP-3 view (Farrugia, 1997) of the two molecules A and B in the asymmetric unit of the title compound. Displacement ellipsoids are drawn at the 40% probability level.

**Fig. 2.**
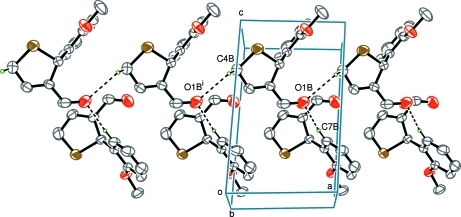
The molecular packing of the title compound showing H-bridge interactions.

## supplementary crystallographic information

### Comment

There has been growing interest in the study of domino or cascade reaction as it allows in principle the formation of multiple new bonds and stereocenters (Enders *et al.*, 2007; Yu & Wang, 2008) in a one-pot system. Consequently, The title compound, (I), was synthesized as one of a series of thio-Michael-aldol products under investigation. In this paper, the absolute configuration and crystal structure of (I) has been presented.

The crystal structure of the title compoud (Fig. 1) contains two independent molecules (molecules A and B) in an asymmetric unit wherein C1A and C1B atoms have been established to exhibit *R* configuration. The main structure unit is a five-membered 2,5-dihydrothiophene ring with an aldehyde group and a 2-methoxyphenyl group. In molecule A, the atom S1A of the five-membered ring lies 0.315 (5)Å from the mean plane of C1A/C2A/C3A/C4A, the atoms O2A, C12A of the methoxy group lie -0.057 (5) and -0.213 (5) Å, respectively, from the benzene ring. The dihedral angel between the main planes of atoms CA1/C2A/C3A/C4A and the benzene ring is 75.45 (5)°. In molecule B, the atom S2B of the five-membered ring lies -0.249 (5)Å from the mean plane of C13B/C14B/C15B/C16B, the atoms O4B, C24B of the methoxy group lie 0.039 (5) and 0.001 (5) Å, respectively, from the benzene ring. The dihedral angel between the main plane formed by the atoms C13B/C14B/C15B/C16B and the benzene ring is 74.10 (5) °. The crystal structure is devoid of any classical hydrogen bonds. However, non-classical intermolecular interactions of the type C—H···O are present in the structure (Table 1).

The crystal structure of 2-morpholino-4-oxo-4,5-dihydrothiophene-3-carbonitrile which is closely related to the title compound has been reported recently (Zhu *et al.*, 2009).

### Experimental

The title compound was prepared by mixing a toluene (1 ml) solution of (*E*)-3-(2-methoxyphenyl)acrylaldehyde (1 mmol) and 1,4-dithiane-2,5-diol (0.6 mmol) in the presence of (*S*)-2-(diphenyl(trimethylsilyloxy)methyl)pyrrolidine (0.2 mmol) as amine catalyst and 4-nitro-benzoic acid (0.1 mmol) as additive at room temperature with stirring. After completion of the reaction, the mixture was washed with water and extracted with ethyl acetate. The solvent was removed under reduced pressure and the residue was purified by silica gel column chromatography (eluent: petroleum ether/diethyl ether). Single crystals were obtained by slow evaporation of an acetone solution.

### Refinement

H atoms were placed in calculated position with C—H = 0.98, 0.97, 0.96 and 0.93 Å  for *sp, sp^2^, sp^3^* and aromatic H-atoms, respectively. All H atoms were included in the final cycles of refinement in riding mode, with *U*_iso_(H)=1.2*U*_eq_ of the carrier atoms.

### Crystal data

**Table:**

C_12_H_12_O_2_S	*Z* = 2
*M**_r_* = 220.28	*F*(000) = 232
Triclinic, *P*1	*D*_x_ = 1.285 Mg m^−^^3^
Hall symbol: P 1	Mo *K*α radiation, λ = 0.71073 Å
*a* = 6.8281 (6) Å	Cell parameters from 4403 reflections
*b* = 7.7624 (9) Å	θ = 3.1–27.4°
*c* = 11.8748 (12) Å	µ = 0.26 mm^−^^1^
α = 71.733 (3)°	*T* = 296 K
β = 79.051 (3)°	Chunk, yellow
γ = 73.488 (3)°	0.46 × 0.42 × 0.38 mm
*V* = 569.50 (10) Å^3^	

### Data collection

**Table:**

Rigaku R-AXIS RAPID diffractometer	4243 independent reflections
Radiation source: rolling anode	3098 reflections with *I* > 2σ(*I*)
graphite	*R*_int_ = 0.031
Detector resolution: 10.00 pixels mm^-1^	θ_max_ = 27.4°, θ_min_ = 3.1°
ω scans	*h* = −7→8
Absorption correction: multi-scan (*ABSCOR*; Higashi, 1995)	*k* = −10→10
*T*_min_ = 0.886, *T*_max_ = 0.905	*l* = −14→15
5623 measured reflections	

### Refinement

**Table:**

Refinement on *F*^2^	Hydrogen site location: inferred from neighbouring sites
Least-squares matrix: full	H-atom parameters constrained
*R*[*F*^2^ > 2σ(*F*^2^)] = 0.039	*w* = 1/[σ^2^(*F*_o_^2^) + (0.0219*P*)^2^ + 0.3733*P*] where *P* = (*F*_o_^2^ + 2*F*_c_^2^)/3
*wR*(*F*^2^) = 0.116	(Δ/σ)_max_ < 0.001
*S* = 1.00	Δρ_max_ = 0.25 e Å^−^^3^
4243 reflections	Δρ_min_ = −0.31 e Å^−^^3^
274 parameters	Extinction correction: *SHELXL97* (Sheldrick, 2008), Fc^*^=kFc[1+0.001xFc^2^λ^3^/sin(2θ)]^-1/4^
3 restraints	Extinction coefficient: 0.053 (5)
Primary atom site location: structure-invariant direct methods	Absolute structure: Flack (1983), 1657 Friedel pairs
Secondary atom site location: difference Fourier map	Flack parameter: 0.02 (9)

### Special details

**Table:**

Geometry. All e.s.d.'s (except the e.s.d. in the dihedral angle between two l.s. planes) are estimated using the full covariance matrix. The cell e.s.d.'s are taken into account individually in the estimation of e.s.d.'s in distances, angles and torsion angles; correlations between e.s.d.'s in cell parameters are only used when they are defined by crystal symmetry. An approximate (isotropic) treatment of cell e.s.d.'s is used for estimating e.s.d.'s involving l.s. planes.
Refinement. Refinement of *F*^2^ against ALL reflections. The weighted *R*-factor *wR* and goodness of fit *S* are based on *F*^2^, conventional *R*-factors *R* are based on *F*, with *F* set to zero for negative *F*^2^. The threshold expression of *F*^2^ > σ(*F*^2^) is used only for calculating *R*-factors(gt) *etc*. and is not relevant to the choice of reflections for refinement. *R*-factors based on *F*^2^ are statistically about twice as large as those based on *F*, and *R*- factors based on ALL data will be even larger.

### Fractional atomic coordinates and isotropic or equivalent isotropic displacement parameters (Å^2^)

**Table:**

	*x*	*y*	*z*	*U*_iso_*/*U*_eq_	
S1B	0.18231 (16)	−0.06575 (15)	0.81497 (10)	0.0748 (4)	
S1A	0.48209 (17)	1.01438 (17)	0.33170 (10)	0.0809 (4)	
O2A	0.9679 (4)	1.0496 (4)	0.2171 (2)	0.0612 (7)	
C11B	0.5790 (6)	0.0081 (5)	0.9189 (3)	0.0524 (9)	
C6A	0.8721 (5)	0.7749 (5)	0.3322 (3)	0.0461 (8)	
C11A	1.0032 (5)	0.8609 (5)	0.2381 (3)	0.0475 (8)	
C1B	0.3932 (5)	0.0505 (5)	0.7461 (3)	0.0499 (8)	
H1B	0.5127	−0.0423	0.7236	0.060*	
C6B	0.4530 (5)	0.1295 (5)	0.8313 (3)	0.0470 (8)	
O2B	0.6306 (4)	−0.1755 (4)	0.9175 (3)	0.0719 (8)	
C1A	0.7167 (5)	0.8901 (5)	0.4051 (3)	0.0505 (8)	
H1A	0.7784	0.9821	0.4165	0.061*	
C7A	0.8916 (6)	0.5850 (5)	0.3545 (3)	0.0570 (9)	
H7A	0.8034	0.5268	0.4145	0.068*	
O1B	0.6367 (5)	0.2578 (6)	0.5468 (3)	0.0955 (11)	
C9A	1.1709 (7)	0.5657 (7)	0.2007 (4)	0.0748 (13)	
H9A	1.2740	0.4944	0.1590	0.090*	
C10A	1.1518 (6)	0.7556 (6)	0.1729 (3)	0.0617 (10)	
H10A	1.2380	0.8127	0.1109	0.074*	
C8B	0.4500 (7)	0.3803 (7)	0.9109 (4)	0.0698 (12)	
H8B	0.4055	0.5060	0.9090	0.084*	
O1A	0.9507 (5)	0.6890 (5)	0.6125 (3)	0.0903 (10)	
C5A	0.7736 (7)	0.6832 (6)	0.6196 (4)	0.0682 (11)	
H5A	0.7184	0.6122	0.6908	0.082*	
C9B	0.5748 (7)	0.2607 (7)	0.9928 (4)	0.0726 (12)	
H9B	0.6177	0.3058	1.0455	0.087*	
C3B	0.1198 (6)	0.2121 (6)	0.6214 (4)	0.0707 (12)	
H3B	0.0611	0.2978	0.5550	0.085*	
C2B	0.3153 (5)	0.1889 (5)	0.6343 (3)	0.0539 (9)	
C10B	0.6390 (6)	0.0731 (6)	0.9991 (3)	0.0646 (11)	
H10B	0.7220	−0.0087	1.0569	0.078*	
C7B	0.3878 (6)	0.3165 (5)	0.8297 (3)	0.0584 (9)	
H7B	0.3020	0.3997	0.7738	0.070*	
C2A	0.6391 (6)	0.7799 (5)	0.5245 (3)	0.0525 (9)	
C3A	0.4430 (6)	0.7717 (6)	0.5405 (4)	0.0636 (11)	
H3A	0.3857	0.7042	0.6124	0.076*	
C4B	−0.0019 (7)	0.0973 (8)	0.7161 (4)	0.0938 (17)	
H4B1	−0.0666	0.0312	0.6826	0.113*	
H4B2	−0.1076	0.1748	0.7585	0.113*	
C8A	1.0396 (7)	0.4804 (6)	0.2892 (4)	0.0709 (12)	
H8A	1.0503	0.3530	0.3051	0.085*	
C12A	1.0829 (7)	1.1464 (6)	0.1151 (4)	0.0725 (12)	
H12A	1.2258	1.1087	0.1270	0.109*	
H12B	1.0340	1.2787	0.1044	0.109*	
H12C	1.0661	1.1167	0.0454	0.109*	
C5B	0.4545 (8)	0.2860 (6)	0.5426 (4)	0.0720 (12)	
H5B	0.3984	0.3757	0.4767	0.086*	
C4A	0.3209 (7)	0.8746 (7)	0.4393 (4)	0.0794 (14)	
H4A1	0.1932	0.9538	0.4647	0.095*	
H4A2	0.2893	0.7886	0.4052	0.095*	
C12B	0.7650 (8)	−0.3065 (7)	1.0013 (5)	0.0974 (18)	
H12D	0.8876	−0.2644	0.9932	0.146*	
H12E	0.8005	−0.4266	0.9861	0.146*	
H12F	0.6971	−0.3162	1.0808	0.146*	

### Atomic displacement parameters (Å^2^)

**Table:**

	*U*^11^	*U*^22^	*U*^33^	*U*^12^	*U*^13^	*U*^23^
S1B	0.0757 (7)	0.0954 (8)	0.0581 (6)	−0.0439 (6)	−0.0105 (5)	−0.0051 (6)
S1A	0.0595 (6)	0.0968 (9)	0.0570 (6)	0.0082 (5)	−0.0066 (5)	−0.0044 (6)
O2A	0.0662 (17)	0.0593 (16)	0.0527 (15)	−0.0238 (13)	0.0090 (12)	−0.0094 (13)
C11B	0.052 (2)	0.059 (2)	0.0457 (19)	−0.0200 (17)	−0.0097 (15)	−0.0055 (16)
C6A	0.0444 (18)	0.0550 (19)	0.0367 (16)	−0.0105 (14)	−0.0069 (13)	−0.0094 (14)
C11A	0.0433 (19)	0.054 (2)	0.0402 (17)	−0.0074 (14)	−0.0049 (13)	−0.0092 (15)
C1B	0.0415 (19)	0.058 (2)	0.0439 (18)	−0.0088 (15)	−0.0053 (14)	−0.0082 (16)
C6B	0.0435 (19)	0.057 (2)	0.0398 (17)	−0.0143 (14)	−0.0025 (13)	−0.0124 (15)
O2B	0.077 (2)	0.0537 (16)	0.082 (2)	−0.0061 (13)	−0.0380 (15)	−0.0055 (14)
C1A	0.0468 (19)	0.055 (2)	0.0415 (18)	−0.0078 (15)	0.0016 (14)	−0.0100 (15)
C7A	0.066 (2)	0.056 (2)	0.0419 (18)	−0.0080 (17)	−0.0076 (16)	−0.0094 (16)
O1B	0.081 (3)	0.134 (3)	0.072 (2)	−0.059 (2)	0.0003 (18)	−0.006 (2)
C9A	0.069 (3)	0.084 (3)	0.062 (3)	0.010 (2)	−0.003 (2)	−0.034 (2)
C10A	0.050 (2)	0.083 (3)	0.047 (2)	−0.0103 (19)	0.0017 (16)	−0.021 (2)
C8B	0.083 (3)	0.067 (3)	0.066 (3)	−0.030 (2)	0.010 (2)	−0.026 (2)
O1A	0.077 (2)	0.115 (3)	0.080 (2)	−0.0127 (19)	−0.0280 (18)	−0.027 (2)
C5A	0.074 (3)	0.081 (3)	0.045 (2)	−0.016 (2)	−0.0056 (19)	−0.015 (2)
C9B	0.080 (3)	0.098 (4)	0.057 (2)	−0.046 (3)	0.005 (2)	−0.031 (2)
C3B	0.057 (3)	0.093 (3)	0.048 (2)	−0.001 (2)	−0.0129 (18)	−0.010 (2)
C2B	0.052 (2)	0.064 (2)	0.0389 (18)	−0.0054 (16)	−0.0100 (15)	−0.0095 (16)
C10B	0.060 (2)	0.093 (3)	0.047 (2)	−0.031 (2)	−0.0098 (17)	−0.013 (2)
C7B	0.066 (2)	0.058 (2)	0.049 (2)	−0.0163 (18)	−0.0009 (17)	−0.0139 (17)
C2A	0.056 (2)	0.058 (2)	0.0421 (19)	−0.0149 (16)	0.0033 (15)	−0.0157 (16)
C3A	0.061 (3)	0.079 (3)	0.050 (2)	−0.026 (2)	0.0066 (18)	−0.017 (2)
C4B	0.056 (3)	0.150 (5)	0.068 (3)	−0.029 (3)	−0.007 (2)	−0.015 (3)
C8A	0.088 (3)	0.061 (2)	0.052 (2)	0.005 (2)	−0.008 (2)	−0.019 (2)
C12A	0.076 (3)	0.087 (3)	0.054 (2)	−0.042 (2)	0.007 (2)	−0.007 (2)
C5B	0.090 (4)	0.078 (3)	0.044 (2)	−0.027 (2)	−0.007 (2)	−0.0043 (19)
C4A	0.061 (3)	0.107 (4)	0.076 (3)	−0.022 (2)	−0.011 (2)	−0.030 (3)
C12B	0.091 (4)	0.074 (3)	0.116 (4)	−0.021 (3)	−0.058 (3)	0.017 (3)

### Geometric parameters (Å, °)

**Table:**

S1B—C4B	1.813 (5)	C8B—C7B	1.389 (6)
S1B—C1B	1.839 (4)	C8B—H8B	0.9300
S1A—C4A	1.816 (5)	O1A—C5A	1.209 (5)
S1A—C1A	1.839 (4)	C5A—C2A	1.468 (6)
O2A—C11A	1.363 (4)	C5A—H5A	0.9300
O2A—C12A	1.433 (4)	C9B—C10B	1.379 (6)
C11B—O2B	1.372 (5)	C9B—H9B	0.9300
C11B—C10B	1.377 (5)	C3B—C2B	1.327 (5)
C11B—C6B	1.405 (5)	C3B—C4B	1.478 (6)
C6A—C7A	1.385 (5)	C3B—H3B	0.9300
C6A—C11A	1.410 (4)	C2B—C5B	1.469 (6)
C6A—C1A	1.507 (5)	C10B—H10B	0.9300
C11A—C10A	1.387 (5)	C7B—H7B	0.9300
C1B—C2B	1.500 (4)	C2A—C3A	1.333 (5)
C1B—C6B	1.502 (5)	C3A—C4A	1.476 (6)
C1B—H1B	0.9800	C3A—H3A	0.9300
C6B—C7B	1.387 (5)	C4B—H4B1	0.9700
O2B—C12B	1.431 (5)	C4B—H4B2	0.9700
C1A—C2A	1.495 (4)	C8A—H8A	0.9300
C1A—H1A	0.9800	C12A—H12A	0.9600
C7A—C8A	1.383 (5)	C12A—H12B	0.9600
C7A—H7A	0.9300	C12A—H12C	0.9600
O1B—C5B	1.208 (5)	C5B—H5B	0.9300
C9A—C8A	1.375 (6)	C4A—H4A1	0.9700
C9A—C10A	1.379 (6)	C4A—H4A2	0.9700
C9A—H9A	0.9300	C12B—H12D	0.9600
C10A—H10A	0.9300	C12B—H12E	0.9600
C8B—C9B	1.354 (7)	C12B—H12F	0.9600
			
C4B—S1B—C1B	95.1 (2)	C2B—C3B—H3B	121.1
C4A—S1A—C1A	94.67 (19)	C4B—C3B—H3B	121.1
C11A—O2A—C12A	117.2 (3)	C3B—C2B—C5B	122.7 (4)
O2B—C11B—C10B	124.6 (3)	C3B—C2B—C1B	116.8 (3)
O2B—C11B—C6B	114.3 (3)	C5B—C2B—C1B	120.4 (3)
C10B—C11B—C6B	121.1 (3)	C11B—C10B—C9B	119.3 (4)
C7A—C6A—C11A	118.2 (3)	C11B—C10B—H10B	120.3
C7A—C6A—C1A	122.1 (3)	C9B—C10B—H10B	120.3
C11A—C6A—C1A	119.7 (3)	C6B—C7B—C8B	120.4 (4)
O2A—C11A—C10A	124.6 (3)	C6B—C7B—H7B	119.8
O2A—C11A—C6A	115.0 (3)	C8B—C7B—H7B	119.8
C10A—C11A—C6A	120.4 (3)	C3A—C2A—C5A	121.4 (4)
C2B—C1B—C6B	116.1 (3)	C3A—C2A—C1A	117.2 (4)
C2B—C1B—S1B	103.9 (3)	C5A—C2A—C1A	121.5 (3)
C6B—C1B—S1B	111.5 (2)	C2A—C3A—C4A	117.0 (4)
C2B—C1B—H1B	108.4	C2A—C3A—H3A	121.5
C6B—C1B—H1B	108.4	C4A—C3A—H3A	121.5
S1B—C1B—H1B	108.4	C3B—C4B—S1B	105.0 (3)
C7B—C6B—C11B	117.8 (3)	C3B—C4B—H4B1	110.7
C7B—C6B—C1B	123.5 (3)	S1B—C4B—H4B1	110.7
C11B—C6B—C1B	118.7 (3)	C3B—C4B—H4B2	110.7
C11B—O2B—C12B	117.5 (4)	S1B—C4B—H4B2	110.7
C2A—C1A—C6A	114.7 (3)	H4B1—C4B—H4B2	108.8
C2A—C1A—S1A	103.7 (2)	C9A—C8A—C7A	119.6 (4)
C6A—C1A—S1A	112.1 (3)	C9A—C8A—H8A	120.2
C2A—C1A—H1A	108.7	C7A—C8A—H8A	120.2
C6A—C1A—H1A	108.7	O2A—C12A—H12A	109.5
S1A—C1A—H1A	108.7	O2A—C12A—H12B	109.5
C8A—C7A—C6A	121.3 (4)	H12A—C12A—H12B	109.5
C8A—C7A—H7A	119.4	O2A—C12A—H12C	109.5
C6A—C7A—H7A	119.4	H12A—C12A—H12C	109.5
C8A—C9A—C10A	120.9 (4)	H12B—C12A—H12C	109.5
C8A—C9A—H9A	119.6	O1B—C5B—C2B	124.7 (4)
C10A—C9A—H9A	119.6	O1B—C5B—H5B	117.7
C9A—C10A—C11A	119.6 (4)	C2B—C5B—H5B	117.7
C9A—C10A—H10A	120.2	C3A—C4A—S1A	105.1 (3)
C11A—C10A—H10A	120.2	C3A—C4A—H4A1	110.7
C9B—C8B—C7B	120.4 (4)	S1A—C4A—H4A1	110.7
C9B—C8B—H8B	119.8	C3A—C4A—H4A2	110.7
C7B—C8B—H8B	119.8	S1A—C4A—H4A2	110.7
O1A—C5A—C2A	124.9 (4)	H4A1—C4A—H4A2	108.8
O1A—C5A—H5A	117.6	O2B—C12B—H12D	109.5
C2A—C5A—H5A	117.6	O2B—C12B—H12E	109.5
C8B—C9B—C10B	120.8 (4)	H12D—C12B—H12E	109.5
C8B—C9B—H9B	119.6	O2B—C12B—H12F	109.5
C10B—C9B—H9B	119.6	H12D—C12B—H12F	109.5
C2B—C3B—C4B	117.9 (4)	H12E—C12B—H12F	109.5
			
C12A—O2A—C11A—C10A	4.8 (5)	C7B—C8B—C9B—C10B	−1.4 (7)
C12A—O2A—C11A—C6A	−174.0 (3)	C4B—C3B—C2B—C5B	176.0 (4)
C7A—C6A—C11A—O2A	176.6 (3)	C4B—C3B—C2B—C1B	−1.4 (6)
C1A—C6A—C11A—O2A	−3.4 (4)	C6B—C1B—C2B—C3B	−113.9 (4)
C7A—C6A—C11A—C10A	−2.2 (5)	S1B—C1B—C2B—C3B	8.8 (4)
C1A—C6A—C11A—C10A	177.7 (3)	C6B—C1B—C2B—C5B	68.6 (5)
C4B—S1B—C1B—C2B	−10.7 (3)	S1B—C1B—C2B—C5B	−168.6 (3)
C4B—S1B—C1B—C6B	115.0 (3)	O2B—C11B—C10B—C9B	−179.0 (4)
O2B—C11B—C6B—C7B	177.7 (3)	C6B—C11B—C10B—C9B	−0.3 (5)
C10B—C11B—C6B—C7B	−1.2 (5)	C8B—C9B—C10B—C11B	1.6 (6)
O2B—C11B—C6B—C1B	−1.3 (4)	C11B—C6B—C7B—C8B	1.3 (5)
C10B—C11B—C6B—C1B	179.8 (3)	C1B—C6B—C7B—C8B	−179.7 (3)
C2B—C1B—C6B—C7B	18.9 (5)	C9B—C8B—C7B—C6B	−0.1 (6)
S1B—C1B—C6B—C7B	−99.7 (3)	O1A—C5A—C2A—C3A	−177.7 (5)
C2B—C1B—C6B—C11B	−162.2 (3)	O1A—C5A—C2A—C1A	2.9 (7)
S1B—C1B—C6B—C11B	79.2 (3)	C6A—C1A—C2A—C3A	−111.8 (4)
C10B—C11B—O2B—C12B	−3.4 (6)	S1A—C1A—C2A—C3A	10.7 (4)
C6B—C11B—O2B—C12B	177.7 (4)	C6A—C1A—C2A—C5A	67.6 (5)
C7A—C6A—C1A—C2A	20.1 (5)	S1A—C1A—C2A—C5A	−169.9 (3)
C11A—C6A—C1A—C2A	−159.9 (3)	C5A—C2A—C3A—C4A	179.7 (4)
C7A—C6A—C1A—S1A	−97.8 (3)	C1A—C2A—C3A—C4A	−0.8 (6)
C11A—C6A—C1A—S1A	82.3 (4)	C2B—C3B—C4B—S1B	−6.9 (6)
C4A—S1A—C1A—C2A	−13.5 (3)	C1B—S1B—C4B—C3B	10.1 (4)
C4A—S1A—C1A—C6A	110.7 (3)	C10A—C9A—C8A—C7A	−2.6 (7)
C11A—C6A—C7A—C8A	2.1 (5)	C6A—C7A—C8A—C9A	0.3 (6)
C1A—C6A—C7A—C8A	−177.9 (4)	C3B—C2B—C5B—O1B	−173.9 (5)
C8A—C9A—C10A—C11A	2.5 (7)	C1B—C2B—C5B—O1B	3.4 (7)
O2A—C11A—C10A—C9A	−178.7 (4)	C2A—C3A—C4A—S1A	−9.7 (5)
C6A—C11A—C10A—C9A	0.0 (5)	C1A—S1A—C4A—C3A	13.3 (4)

### Hydrogen-bond geometry (Å, °)

**Table:**

*D*—H···*A*	*D*—H	H···*A*	*D*···*A*	*D*—H···*A*
C7A—H7A···O1B	0.93	2.60	3.473 (4)	157
C4B—H4B1···O1B^i^	0.97	2.69	3.202 (4)	113

Symmetry codes: (i) *x*−1, *y*, *z*.
